# Three-Point Bending Fracture Behavior of Single Oriented Crossed-Lamellar Structure in *Scapharca broughtonii* Shell

**DOI:** 10.3390/ma8095298

**Published:** 2015-09-15

**Authors:** Hong-Mei Ji, Wen-Qian Zhang, Xu Wang, Xiao-Wu Li

**Affiliations:** 1 Institute of Materials Physics and Chemistry, College of Sciences, Northeastern University, Shenyang 110819, China; E-Mails: jhm19900118@sina.com (H.-M.J.); zhangwqneu@yahoo.com (W.-Q.Z.); wangxu55@yahoo.com (X.W.); 2 Key Laboratory for Anisotropy and Texture Engineering of Materials, Ministry of Education, Northeastern University, Shenyang 110819, China

**Keywords:** *Scapharca broughtonii* shell, crossed-lamellar structure, three-point bending, strength, fracture

## Abstract

The three-point bending strength and fracture behavior of single oriented crossed-lamellar structure in *Scapharca broughtonii* shell were investigated. The samples for bending tests were prepared with two different orientations perpendicular and parallel to the radial ribs of the shell, which corresponds to the tiled and stacked directions of the first-order lamellae, respectively. The bending strength in the tiled direction is approximately 60% higher than that in the stacked direction, primarily because the regularly staggered arrangement of the second-order lamellae in the tiled direction can effectively hinder the crack propagation, whereas the cracks can easily propagate along the interfaces between lamellae in the stacked direction.

## 1. Introduction

Over billions of years of evolution, biological shells have been highly mineralized to develop into the materials with outstanding mechanical performances. Despite the fact that most of these materials consist of ordinary inorganic calcium carbonate (CaCO_3_) and biological macromolecules, their mechanical properties are far superior to those for the single crystals of the pure mineral [1,2,3,4,5]. 

As is well known, the crossed-lamellar structure, which is the most common structure in classes Gastropod and Bivalvia [2,6,7], is renewed for its fantastic micro-architecture and corresponding excellent mechanical properties. Particularly, the coarsest structures of shells including several macrolayers arranged in either “weak” or “tough” orientations with respect to the potential crack growth direction, have drawn a great deal of attention in recent years [2,8,9,10,11,12,13]. For instance, in *Strombus gigas* shells with three macrolayers, the crossed-lamellar structures in inner, middle and outer layers are arranged in a 0°/90°/0° mode, meaning that the overall arrangements of the lamellae in the middle macrolayer have an 90° rotation about the axis perpendicular to the outer surface of shell to those in the inner and outer layers. The results show that the strength is higher along the orientation parallel to the spiral axis than that perpendicular to the spiral axis under both uniaxial compression and three-point bending tests [10,12]. Fracturing in the “weak” and “tough” layers have been quantitatively understood by different energy-dissipating mechanisms in *Strombus gigas* shells, *i.e.*, crack bridging and microcracking in “tough” layer account for a larger portion of the dissipated energy, while multiply-channel cracking in the “weak” layer just absorbs a small amount of energy [2,8,9,11]. Recently, we also performed compressive tests on *Veined rapa whelk* shell with two macrolayers (the arrangement of crossed-lamellar structures are mutually perpendicular in these two layers) using four kinds of specimens with loading axis making different angles (α = 0°, 30°, 60° and 90°) with the spiral lines. It was found that the interfaces of different-level lamellae in the adjacent macro-layers yield significant effects on the mechanical behavior in a coordinated fashion [13]. 

There are also some shells, in which the crossed-lamellar structure is oriented merely in one orientation (herein called single oriented crossed-lamellar structure) [14,15,16,17,18]. However, there is much less information about the fracture mechanisms of single oriented crossed-lamellar structure. Thus, in the present work, the three-point bending fracture behavior of *Scapharca broughtonii* shell samples with only one macrolayer was investigated. It is expected that these studies can further reveal the fracture mechanisms in the crossed-lamellar structure, and provide a theoretical basis for developing biomimetic materials.

## 2. Experimental Section

**Figure 1:**
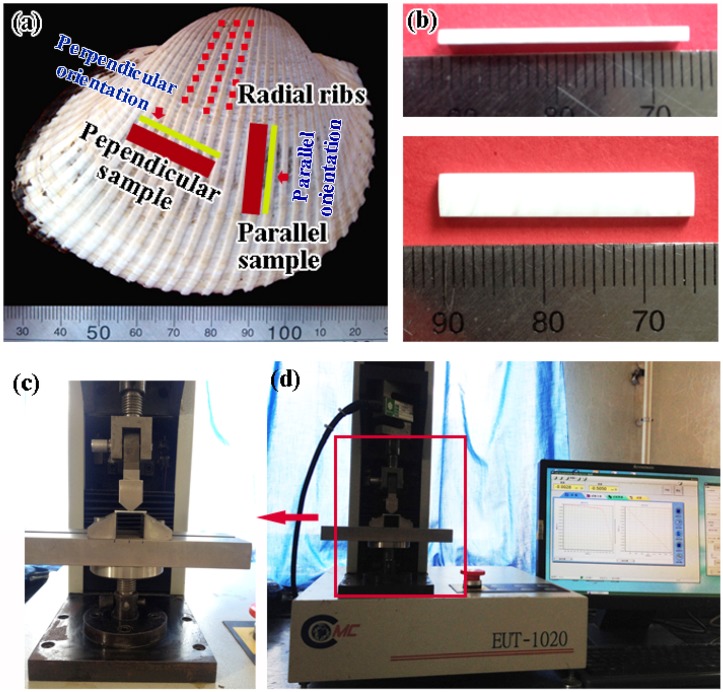
(**a**) Overall view of *Scapharca broughtonii* shell; (**b**) dimensions of sample for three-point bending test, and (**c**,**d**) the experimental equipment for three-point bending tests.

*Scapharca broughtonii* shell, which is a member of the cardiidae family of the Bivalvia class, was adopted as the target material in the present work. The shell was dried at room temperature for several days. As shown in Figure 1a, many obvious radial ribs are arranged on the outer surface of this shell. All investigated specimens were cut from the middle part of this shell.

The directly broken cross-sectional specimens (Figure 1a) with the fracture surfaces perpendicular or parallel to the radial ribs (thereafter named as perpendicular or parallel orientations) were prepared for scanning electron microscope (SEM) (Carl Zeiss, Jena, Germany) observations. 

Specimens for three-point bending tests were firstly cut with a water-cooled low-speed diamond saw, and then grounded carefully with emery papers from 600 # to 3000 #. Figure 1b shows the dimensions (4.5 mm × 1.5 mm × 25 mm) of the samples, and the samples with the long side perpendicular and parallel to the radial ribs are, herein, named as perpendicular and parallel samples respectively, as marked in Figure 1a. The three-point bending tests were conducted under a constant loading rate of 5 × 10^−3^ mm/s with a loading span of 20 mm on Care EUT-1020 testing machine (with a maximum load of 2000 N and an accuracy of 10^−3^ kN) (Care, Tianjin, China), as shown in Figure 1c,d. The bending strength σ*_bb_* was calculated using the common flexure equation [19]:

(1) $${\text{σ}}_{bb}=3PL/2bh^{2}$$

where *P* is the maximum load, *L* the loading span, and *b* and *h* are the width and thickness of samples, respectively.

The obtained bending strengths were further analyzed by means of the Weibull Equation [20]:

(2) $$F(V)=1-\exp[-{\left({\text{σ}}_{bb}/{\text{σ}}_{0}\right)}^{m}]$$

where *F*(*V*) is the failure probability, *m* the Weibull modulus, and σ_0_ is the characteristic strength. 

## 3. Results and Discussion

**Figure 2:**
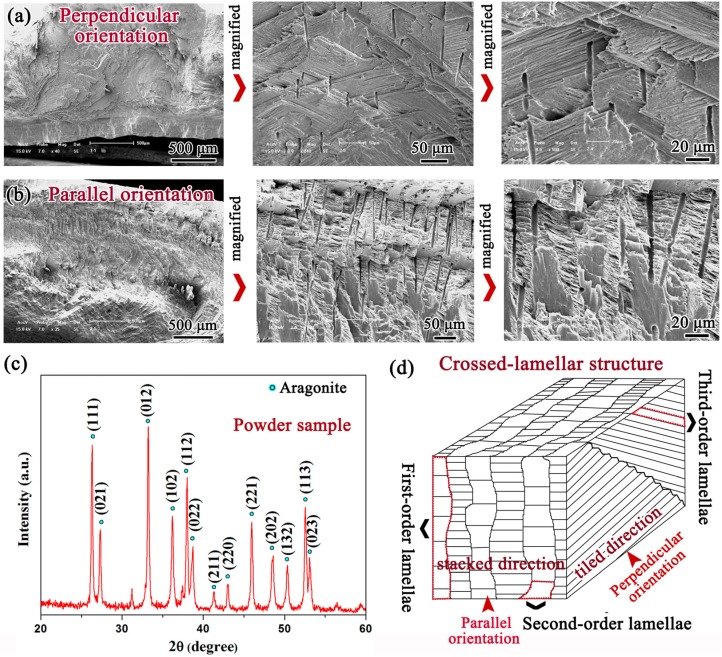
SEM micrographs of cross sections perpendicular (**a**) and parallel (**b**) to the radial ribs; (**c**) XRD patterns of the shell; (**d**) Schematic illustration of the crossed-lamellar structure in the shell.

The SEM images of the cross sections perpendicular and parallel to the radial ribs of *Scapharca broughtonii* shell are shown in Figure 2a,b, respectively. Obviously, most of areas exhibit a crossed-lamellar structure comprising the inorganic phase of aragonite CaCO_3_, as demonstrated by the X-ray diffraction (XRD) (PANalytical, Almelo, The Netherlands) analysis in Figure 2c. It is well known that the crossed-lamellar structure can be divided into three-order lamellae, *i.e.*, the first-order lamella is composed of the second-order lamella, which consists further of the third-order lamella [2,8,9,10,11,12,13,14,15,16,17,18]. The stereoscopic schematic diagram presented in Figure 2d visually illustrates the architecture of the crossed-lamellar structure in *Scapharca broughtonii* shell, and it is clearly seen that the edges of the first-order lamellae are not straight. It is interesting to note that the first-order lamellae are piled up along the parallel orientation (herein called stacked direction), while the first-order lamellae are tiled along the perpendicular orientation (herein called tiled direction).

**Figure 3:**
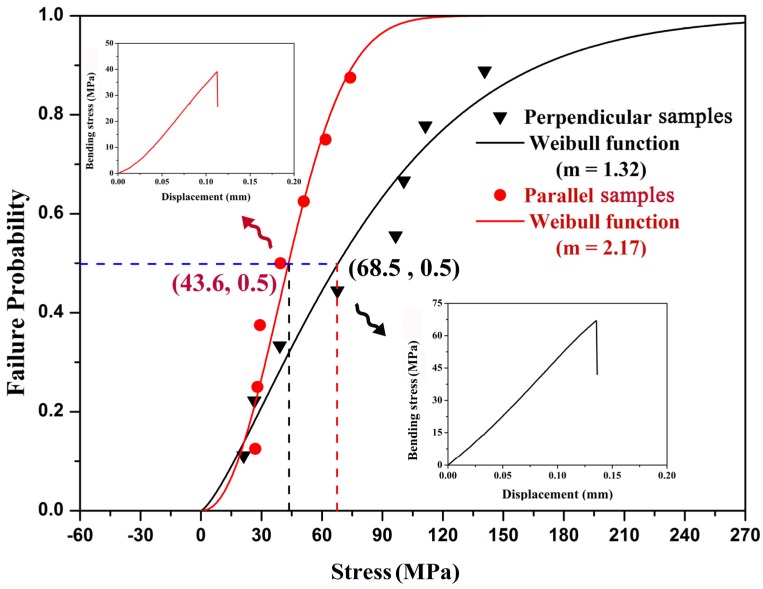
Representative bending stress—displacement curves and Weibull plots of bending strength of *Scapharca broughtonii* shell samples with two directions under three-point bending tests.

Figure 3 shows the representative stress—displacement curves and Weibull functions of the bending tests perpendicular and parallel to the radial ribs, respectively. It can be seen that the 50% fracture probabilities (*F*(*V*) = 50%) of the bending tests are equal to 68.5 ± 41.5 and 43.6 ± 17.2 MPa for perpendicular and parallel samples, respectively. Thus, this shell is stronger in bending tests with the loading direction perpendicular to the radial ribs, and the mean value of bending stress is significantly higher (~60%) in this orientation than that in parallel orientation, which is attributed to the extremely anisotropic crossed-lamellar structure. For the crossed-lamellar structure in a 0°/90°/0° arrangement, the mean strength of the tested samples is 24.0–74.0 MPa for *Strombus gigas* shell [10], and 107.5–207.0 MPa for *Conus striatus* shell [21]. Therefore, the bending strength is strongly dependent upon the species of shells. However, Currey and Kohn [21] also performed three-point bending tests on *Conus* *miles* shell, which also has three macrolayers with the crossed-lamellar structure in a 0°/90°/0° arrangement. Half of whose test pieces were prepared by grinding off the inner layer, and they found that the mean strength for the *Conus miles* shell samples with three macrolayers is 54.1 MPa, but for those with two maccrolayers it is 194.8 MPa. Thus, the arrangement of the crossed-lamellar structure also plays an important role in the bending mechanical properties.

**Figure 4:**
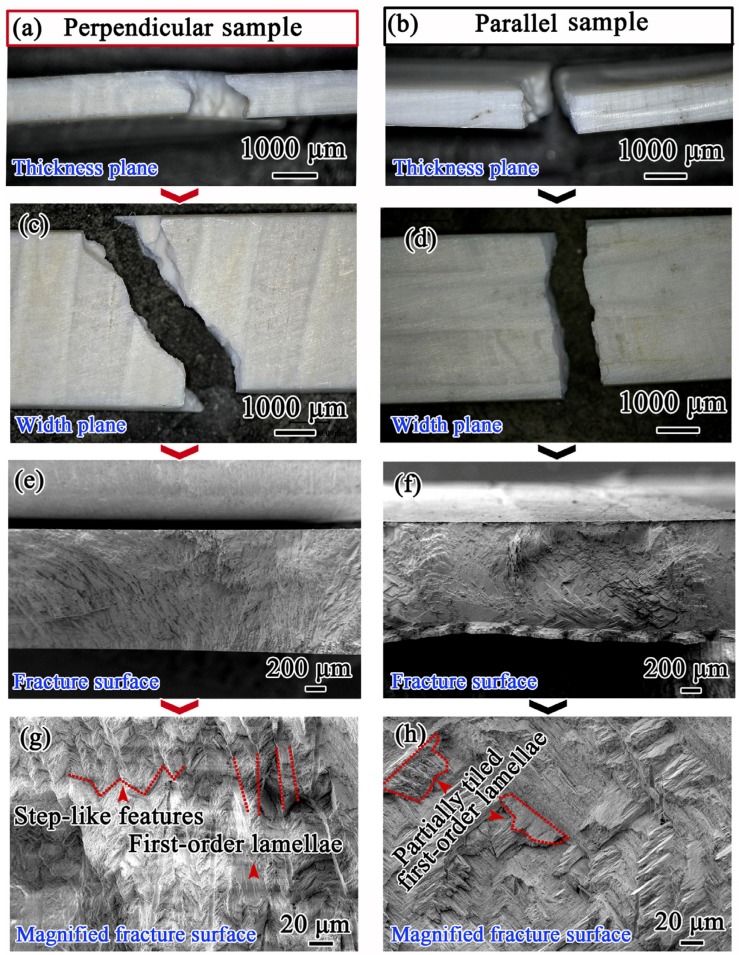
SEM images of the fracture planes of perpendicular (**a**,**c**,**e**,**g**) and parallel (**b**,**d**,**f**,**h**) samples of *Scapharca broughtonii* shell.

Figure 4 gives the fracture surface morphologies observed along different directions of bending samples. The irregular zigzag fracture features are clearly observed both along the thickness and width directions on the perpendicular samples (Figure 4a,c), as compared to those for parallel samples (Figure 4b,d). From Figure 4e,g, it can be detected that the fracture pattern is quite rough, and the fracture paths between first-order lamellae show evidently step-like features in perpendicular samples. In contrast, the fracture surface is relatively smooth, and partially tiled first-order lamellae comprising successive third-order lamellae are obviously presented in parallel samples, as indicated in Figure 4f,h. 

**Figure 5:**
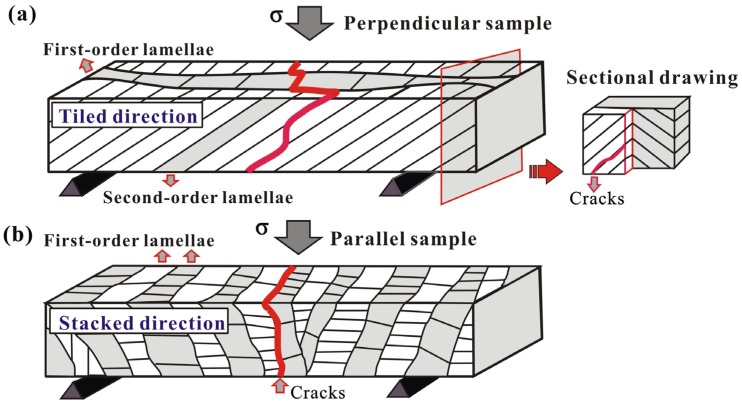
Schematics of the principal fracture mechanisms in *Scapharca broughtonii* shell samples prepared perpendicular (**a**) and parallel (**b**) to the radial ribs.

In *Scapharca broughtonii* shell, considering the potential catastrophic crack propagation direction under three-point bending tests, these perpendicular and parallel samples can be further described as the “tough” and “weak” samples, respectively. Specifically, in the “tough” sample, cracks propagate along the preferred direction, namely, the interfaces between the second-order lamellae, as shown in Figure 5a. However, in the adjacent first-order lamellae, the second-order lamellae are arranged by ± 45° orientation with respect to the loading direction; in this case, cracks propagate first along the preferred direction (interfaces between the second-order lamellae), but they are subsequently arrested by the second-order lamellae perpendicular to the crack propagation in the adjacent first-order lamella (see the sectional drawing in Figure 5a). In contrast, sequential cracking along the weak interface between first-order lamellae occurs during bending deformation in the “weak” orientation (Figure 5b), causing that only a relatively little energy can completely fracture the shell. 

Compared with the fracture behavior of the “weak” layer in *Strombus gigas* shell including three macrolayers [2,8,9,11], there are no channel cracks observed in the current *Scapharca broughtonii* shell. The above experimental results indicate that the energy-dissipating mechanism in *Strombus gigas* shell with three macrolayers suggested by Kessler *et al.* [8] does not apply to the current single oriented crossed-lamellar structure in *Scapharca broughtonii* shell, where channel cracks cannot form in the “weak” orientation during bending deformation. Specifically, in the current *Scapharca broughtonii* shell, the crossed-lamellar structure is oriented in single direction, resulting in the deficiency of channel cracks in “weak” orientation due to lacking of the arrestment of the “tough” layer. Accordingly, the primary fracture resistance (or mechanism) in single oriented crossed-lamellar structure is closely related to the fact that the regularly staggered arrangement of the second-order lamellae among the adjacent first-order lamellae can effectively hinder the crack propagation.

## 4. Conclusions 

*Scapharca broughtonii* shell presents a typical crossed-lamellar structure, which is oriented in single direction. As the three-point bending sample is prepared parallel to the radial ribs, the long axis of sample is exactly the stacked direction of the fist-order lamellae, for which the cracks propagate along the interfaces between lamellae to cause easily a rapid fracture. As the sample is prepared perpendicular to the radial ribs, the long axis of sample is exactly the tiled direction of the fist-order lamellae; in this case, the regularly staggered arrangement of second-order lamellae can effectively block the crack propagation, thus significantly increasing the resistance to fracture. Therefore, the bending strength is significantly higher (~60%) in the perpendicular orientation than that in the parallel orientation.
